# Water-deficit responsive microRNAs in the primary root growth zone of maize

**DOI:** 10.1186/s12870-019-2037-y

**Published:** 2019-10-24

**Authors:** Candace M. Seeve, Ramanjulu Sunkar, Yun Zheng, Li Liu, Zhijie Liu, Michael McMullen, Sven Nelson, Robert E. Sharp, Melvin J. Oliver

**Affiliations:** 1 USDA-ARS, Plant Genetics Research Unit, Columbia, MO 65211 USA; 2 Interdisciplinary Plant Group, University of Missouri, Columbia, MO 65211 USA; 3 Department of Biochemistry and Molecular Biology, Oklahoma State University, Stillwater, OK 74078 USA; 4 Yunnan Key Laboratory of Primate Biomedical Research, Kunming University of Science and Technology, Kunming, 650500 Yunnan China; 5 Faculty of Life Science and Technology, Kunming University of Science and Technology, Kunming, 650500 Yunnan China; 6 Huazhong Agricultural University, Wuhan, 430070 Hubei Province China; 7 Division of Plant Sciences, Columbia, University of Missouri, Columbia, MO 65211 USA; 8 Interdisciplinary Plant Group, University of Missouri, Columbia, MO 65211 USA

**Keywords:** Drought, Water-deficit, microRNA, Small RNA-seq, Degradome, Primary root, Growth zone, Environmental stress, *Zea mays* L

## Abstract

**Background:**

MicroRNA-mediated gene regulatory networks play a significant role in plant growth and development and environmental stress responses.

**Results:**

We identified 79 microRNAs (miRNAs) and multiple miRNA variants (isomiRs) belonging to 26 miRNA families in the primary root growth zone of maize seedlings grown at one of three water potentials: well-watered (− 0.02 MPa), mild water deficit stress (− 0.3 MPa), and severe water deficit stress (− 1.6 MPa). The abundances of 3 miRNAs (mild stress) and 34 miRNAs representing 17 families (severe stress) were significantly different in water-deficit stressed relative to well-watered controls (FDR < 0.05 and validated by stem loop RT-qPCR). Degradome sequencing revealed 213 miRNA-regulated transcripts and trancriptome profiling revealed that the abundance of 77 (miRNA-regulated) were regulated by water-defecit stress. miR399e,i,j-3p was strongly regulated by water-defcit stress implicating the possibility of nutrient deficiency during stress.

**Conclusions:**

We have identified a number of maize miRNAs that respond to specific water deficits applied to the primary root growth zone. We have also identified transcripts that are targets for miRNA regulation in the root growth zone under water-deficit stress. The miR399e,i,j-3p that is known to regulate phosphate uptake in response to nutrient deficiencies responds to water-deficit stress, however, at the seedling stage the seed provides adequate nutrients for root growth thus miR399e,i,j-3p may play a separate role in water-deficit responses. A water-deficit regulated maize transcript, similar to known miR399 target mimics, was identified and we hypothesized that it is another regulatory player, moderating the role of miR399e,i,j-3p, in primary root growth zone water deficit responses.

## Background

Drought is the most important abiotic factor limiting maize productivity globally [1, 2]. Consequently, there is significant interest in identifying the components, from the physiological to the molecular, that contribute to drought tolerance. Tolerance to a soil water deficit involves two mechanisms: avoidance of plant and cellular dehydration, and when dehydration occurs, acclimation to maintain normal growth and reproduction as much as possible [3–5]. Drought-tolerant maize (*Zea mays* L.) hybrids that maintain higher grain yield under water limited conditions do so primarily through dehydration avoidance [2, 3, 6]. Plant physiological adaptations such as a shorter anthesis-silking interval [6], inhibition of shoot growth [7], and changes in root architecture [8–10] facilitate dehydration avoidance by maintaining a more favorable soil-plant water balance throughout growth and development [2, 3, 6]. In a meta-analysis of several physiological adaptations that facilitate dehydration avoidance in maize, Hammer et al. [10] specifically pointed to a larger maize root system architecture as an important contributor for improved yield performance under drought.

Under water deficit conditions, some types of roots possess the ability to continue elongation at low water potentials (Ψ_w_) that completely inhibit shoot growth [11–13]. This capacity is pronounced in the primary root of developing seedlings in a range of species including maize [14, 15], and the physiology of this response has been studied extensively (for review, see [16]). Characterizing the gene regulatory networks that maintain maize root growth under water deficits would provide genetic and biotechnological targets to favorably manipulate a key drought tolerance trait and ultimately improve yield in drought conditions. Gene regulatory networks are comprised of trans-regulatory molecules, including transcription factors (TFs) and regulatory small RNAs, that bind to sequence-specific cis-regulatory elements in or near the genes or transcripts that they regulate [17]. Several studies have focused on identifying TF gene transcripts and genome-wide networks of co-expressed genes that are differentially regulated in the maize primary root growth zone under water deficit stress [5, 15, 18, 19]. However, the expression patterns and roles of regulatory small RNAs in response to water deficit stress in the maize primary root growth zone have not been explored to equal depth.

Among the different classes of regulatory small RNAs, microRNAs (miRNAs) have been recognized as important post-transcriptional trans-regulatory factors in gene regulatory networks controlling development and in response to abiotic stresses [20–22]. Plant microRNAs (miRNAs) are endogenous noncoding RNAs generally 21 nt long that post-transcriptionally regulate target gene expression. Mature miRNAs originate from a longer primary transcript (pri-miRNA) transcribed from the miRNA gene. The pri-miRNA folds imperfectly to form a hairpin loop that is processed by DICER-LIKE enzyme 1, and is released as a double-stranded miRNA/miRNA* duplex. Both strands of the duplex may be incorporated into the RNA-induced silencing complex (RISC) where they regulate complementary target mRNAs, most often by guiding cleavage of the target [23]. Studies in multiple species, including a study that compared miRNA-associated regulatory patterns of two contrasting maize genotypes (drought tolerant vs. sensitive), have found that a large number of miRNAs are differentially expressed under water deficit conditions, and subsequently regulate the expression of other drought-responsive genes [20–22, 24].

To identify miRNAs that are differentially regulated by water deficit stress in the maize seedling primary root growth zone, we used Illumina sequencing to examine the global expression of miRNAs in the 1-cm apical region of the primary root exposed to either a mild ((Ψ_w_ of − 0.3 MPa) or severe (Ψ_w_ of − 1.6 MPa) water deficit stress applied using a vermiculite culture system [14]. We identified putative mRNA targets of water deficit stress-responsive miRNAs, and examined patterns of target transcript abundance under water deficit stress treatments relative to well-watered conditions from degradome and RNA-seq libraries, respectively.

## Results

### Maize seedling primary root growth under water deficit stress

Maize seedlings were grown under mild (Ψ_w_ of − 0.3 MPa), severe (Ψ_w_ of − 1.6 MPa), or no stress/control conditions (Ψ_w_ of − 0.02 MPa) for 26 h. The resulting root growth zone tissue Ψ_w_ were – 0.30 MPa (+/− 0.02) for the − 0.02 MPa treatment, − 0.50 MPa (+/− 0.02) for the − 0.30 MPa treatment, and − 1.64 MPa (+/− 0.04) for the − 1.6 MPa treatment. The growth responses of the primary root grown under water deficit stress were similar to those described previously for B73 in the same system which showed that root growth rates were approximately 38 and 18% of well-watered rates in the mild and severe stress treatments, respectively [5, 25].

### Detection of microRNAs in the primary root growth zone exposed to well-watered or water deficit conditions by small RNA-seq

**Fig. 1:**
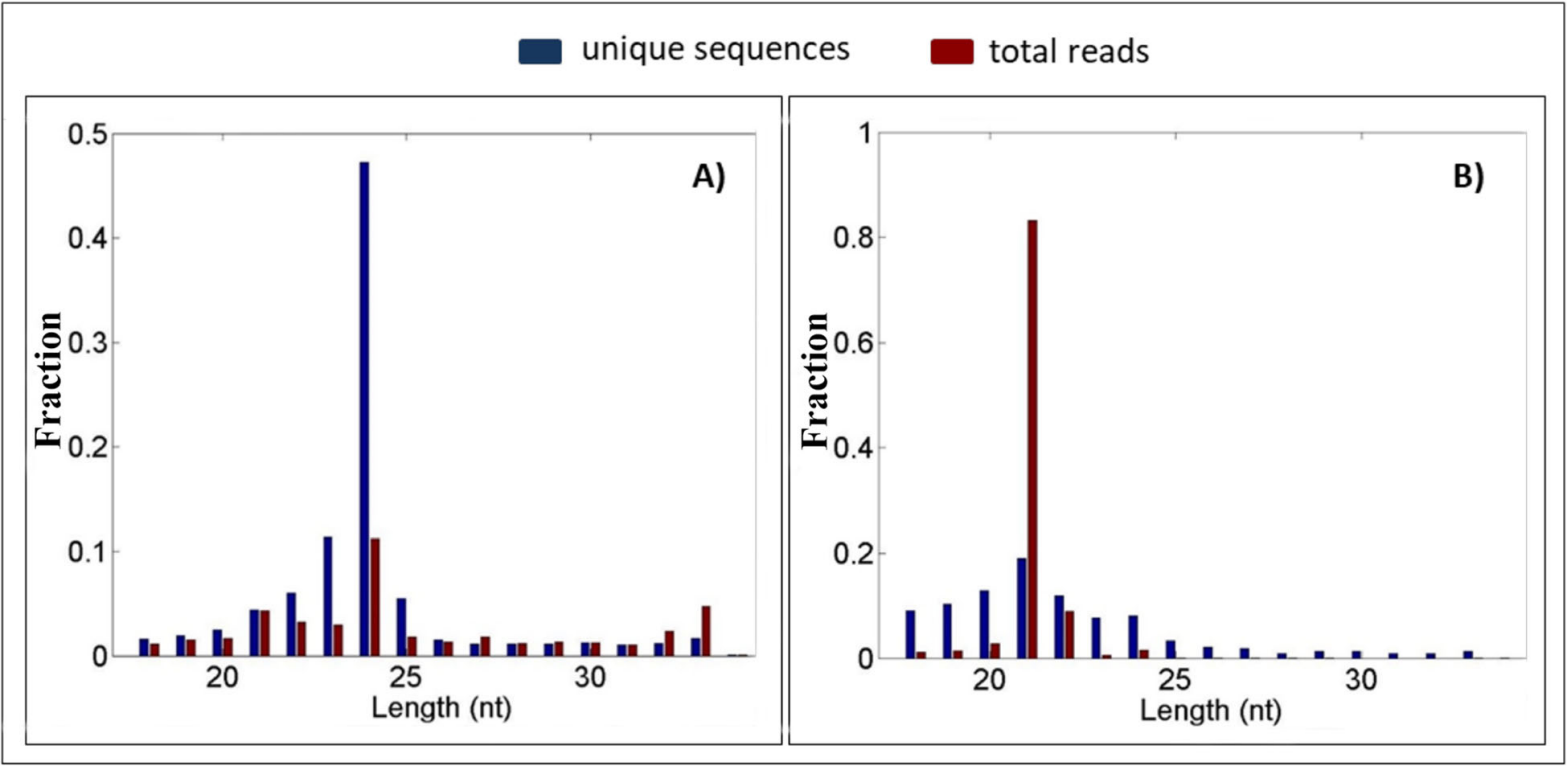
**a** Length distribution of total reads (red bars) and unique sequences (blue bars) mapped to the maize genome. **b** Length distribution of total reads (red bars) and unique sequences (blue bars) mapped to a pre-miRNA annotated in miRBase

The total number of reads generated for each of the 12 libraries ranged from 2,823,122 to 4,990,091 reads (Additional file 3: Table S3). More than 80% of the reads of each library mapped to the maize B73 RefGen_v3 (Release 5b+) genome indicating that the libraries were of high quality (Additional file 3: Table S3). The number of unique reads in each of the twelve libraries ranged from 743,923 to 1,195,411 reads (Additional file 3: Table S3). The small RNA sequences were 21- to 25-nt in size with the largest percentage of sequences that mapped to the maize genome being 24-nt long (Fig. 1 a), and the largest percentage of sequences that aligned to miRbase being 21-nt long (Fig. 1 b). The distribution of small RNA sequence lengths is similar to those reported previously [26–28]. A total of 79 maize miRNAs were identified (Additional file 4: Table S4). For 47 of these, length or sequence variants were detected (Additional file 5: Table S5). The miRNA sequence variants, described as miRNA isoforms (isomiRs), may be generated by modification of the miRNA precursor nucleotide sequence, by imprecise cleavage during miRNA biogenesis, or by nucleotide addition or trimming of the mature miRNA sequence [20, 29–31]. The majority of isomiRs detected perfectly matched the sequence of a reference maize miRNA, but the isomiR sequence had nucleotide variations at the 5′ and/or 3′ ends. A large number of sequence and length variants isomiRs were also detected. These variant isomiRs included sufficient sequence and length variations such that the isomiR sequence did not align perfectly to any maize miRNA locus or other loci (Additional file 5: Table S5).

The miRNAs that were detected in the maize seedling primary root growth zone belonged to 26 different miRNA families (Additional file 4: Table S4). The majority of these miRNA families are known to be conserved widely across the plant kingdom including miR156, miR159, miR160, miR164, miR165/miR166, miR167, miR168, miR169, miR171, miR319, miR390, miR393, miR394, miR395, miR396, miR398, miR399, and miR408 [32–34]. The only highly conserved miRNA family for which members were not detected was miR397. Three families—miR529, miR827, and miR2118—are moderately conserved families that have been detected across several monocot and dicot plant species, and two families—miR444, and miR528—are monocot-specific miRNA families [32–34]. The monocot-specific miR437 family was also absent from the maize seedling primary root growth zone libraries. The remaining miRNA families, miR5139, miR8155, miR8175 and miR894, are either not widely conserved, or their conservation has not been described [32–34].

The abundance of the detected miRNA families varied extensively from fewer than 10 RPTM per sample to more than 300,000 RPTM per sample (Additional file 10: Figure S2). With an average of > 300,000 RPTM per sample, the miR166 family was the most abundantly expressed family and was at least ten times more abundant than any other miRNA family detected in the primary root growth zone (Additional file 10: Figure S2). The miR166 family transcript levels were extremely abundant in the primary root growth zone across all treatments suggesting that miR166 family miRNAs are not solely stress-responsive and likely have essential roles in regulating primary root growth and development. These results are consistent with previous studies exploring miRNA abundances [35] and miR166 function [36–38] in the root system of other plant species. The miR156, miR159, miR168, miR319, and miR396 families were moderately abundant in the maize seedling primary root growth zone with > 1000 RPTM per sample (Additional file 4: Table S4). Several of the highly conserved miRNA families were expressed at low abundances in the primary root growth zone:miR160, miR164, miR167, miR169, miR171, miR390, miR393, miR394, miR395, miR398, miR399, and miR408 (Additional file 4: Table S4).

### Identification of miRNAs that are differentially regulated under water deficit stress

**Fig. 2:**
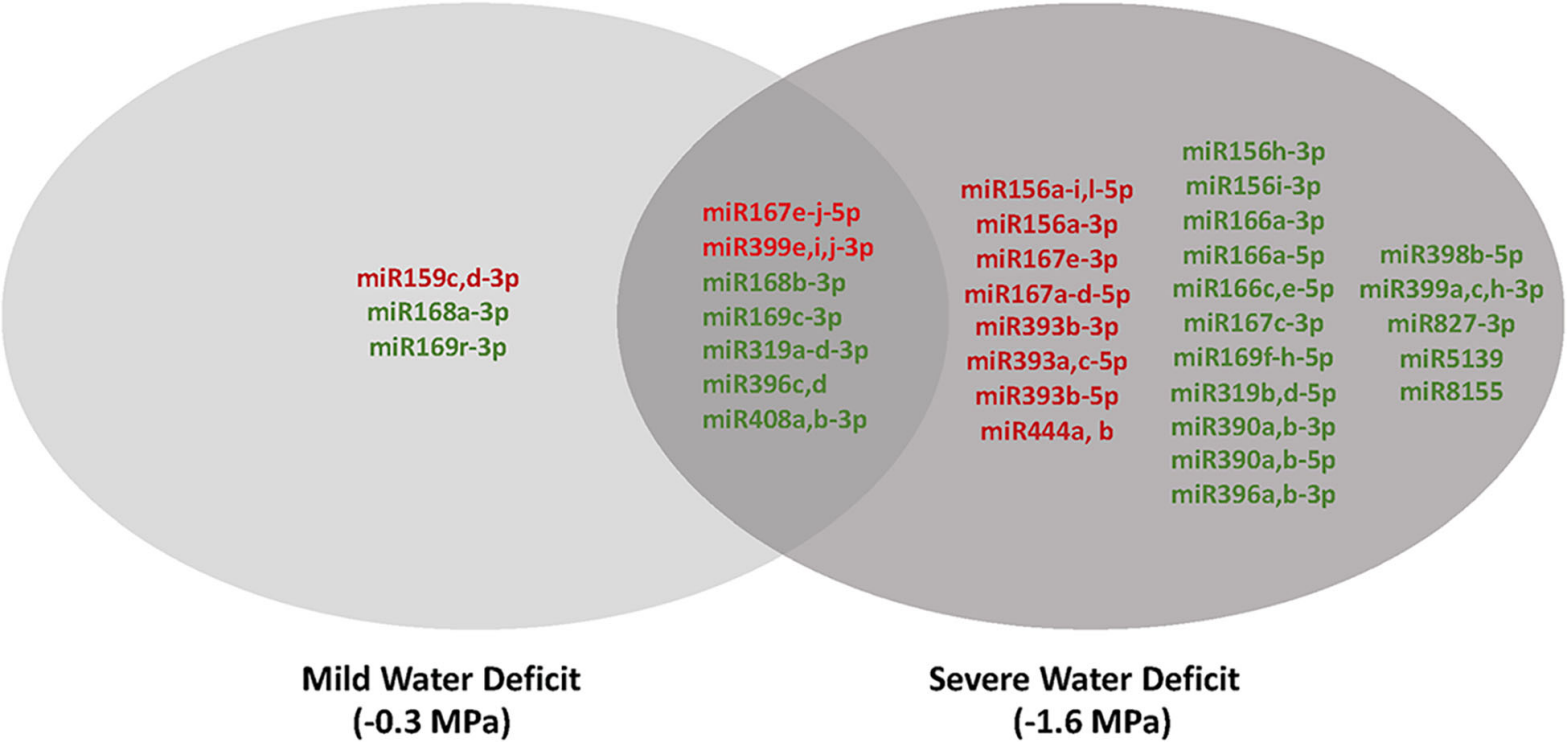
miRNAs for which abundance significantly changed in the maize seedling primary root growth zone under water deficit-stress treatments in comparison to control conditions. Green text indicates that miRNAs were significantly less abundant in the maize seedling primary root growth zone under water deficit-stress than under control conditions. Red text indicates that miRNAs were significantly more abundant in the maize seedling primary root growth zone under water deficit-stress than under control conditions

Among the miRNAs detected in the primary root growth zone, 34 miRNAs were differentially regulated in response to water deficit stress (FDR <0.05) (Table 1). The miRNAs that were differentially regulated by water deficit stress belonged to 17 miRNA families, which represents more than half of the miRNA families that were detected. The large number of differentially regulated miRNAs that were observed indicates that miRNAs likely play a key role(s) in regulating water deficit-induced primary root growth responses. The largest number of miRNAs were differentially regulated in the severe stress treatment only (Fig. 2), whereas only 3 miRNAs were differentially regulated in the mild water deficit stress alone: zma-miR168a-3p, zma-miR169r-3p, zma-miR159c, d-3p. (Fig. 2). None of the differentially-regulated miRNAs were inversely regulated under the two different water deficit stress conditions (Table 1). For the seven miRNAs that were differentially regulated by both mild and severe water deficit stress (Fig. 2), the magnitude of change in transcript abundance was usually similar in both treatments or was smaller in the mild stress treatment than in the severe treatment (Table 1). The exception to this trend was zma-miR399e, i, j-3p for which the magnitude of change in transcript abundance was much larger under mild stress than under severe stress (Table 1).

**Table 1:** List of water deficit stress-regulated miRNAs detected in the primary root growth zone by small RNA-seq.

miRNA ID	WW R2	WW R3	WW R4	WW R5	3R1	3R2	3R3	3R4	log_2_ FC	pvalue	FDR
zma-miR399e, i, j-3p	13	9	7	4	108	80	100	99	3.39	1.1E-20	2.4E-18
zma-miR396c, d	5661	6128	6194	6514	4653	4128	4336	4869	−0.422	7.2E-09	3.2E-07
zma-miR319a-d-3p	5080	5210	5972	4920	3955	3519	3964	4135	−0.422	2.1E-08	7.6E-07
zma-miR168b-3p	252	130	132	138	79	68	89	53	−1.188	9.0E-07	2.4E-05
zma-miR408b-3p, a	405	313	486	418	202	206	186	254	−0.910	8.6E-07	2.4E-05
zma-miR167e-j-5p	301	342	413	365	433	487	558	728	0.653	9.7E-06	1.7E-04
zma-miR168a-3p	218	123	97	152	66	82	86	65	−0.985	4.9E-05	0.001
zma-miR169c-3p	142	101	156	181	92	76	71	84	−0.807	0.001	0.006
zma-miR169r-3p	4	6	0	7	0	0	0	0	−3.78	0.006	0.034
zma-miR159c, d-3p	898	1000	1101	1038	1297	1208	1309	991	0.294	0.008	0.044
miRNA ID	WW R2	WW R3	WW R4	WW R5	16 R1	16 R2	16 R3	16 R4	log_2_ FC	pvalue	FDR
zma-miR396c, d	5661	6128	6194	6514	2446	3064	2597	3271	−0.877	6.7E-32	4.8E-30
zma-miR319a-d-3p	5080	5210	5972	4920	2443	2542	2257	2747	−0.855	1.1E-28	6.0E-27
zma-miR393b-3p	79	120	94	74	355	331	316	376	2.14	2.7E-23	9.6E-22
zma-miR167e-j-5p	301	342	413	365	609	760	736	772	1.26	1.1E-17	2.5E-16
zma-miR390a, b-5p	571	826	944	786	309	274	251	287	−1.23	3.4E-17	6.1E-16
zma-miR393a, c-5p	67	57	73	35	193	257	148	270	2.07	5.8E-17	9.5E-16
zma-miR393b-5p	67	57	73	35	186	257	141	265	2.04	1.9E-16	2.4E-15
zma-miR408b-3p, a	405	313	486	418	75	109	135	142	−1.56	1.7E-15	2.0E-14
zma-miR168b-3p	252	130	132	138	50	66	34	84	−1.34	8.6E-08	8.4E-07
zma-miR319b, d-5p	441	361	465	429	152	251	200	212	−0.832	2.1E-06	1.8E-05
zma-miR166a-3p	313,450	360,388	352,629	343,981	222,704	272,440	228,770	225,716	−0.296	2.5E-05	1.9E-04
zma-miR444a, b	36	35	35	53	50	109	63	86	1.17	3.6E-05	2.6E-04
zma-miR399e, i, j-3p	13	9	7	4	23	17	17	59	1.82	3.8E-05	2.7E-04
zma-miR8155	182	152	292	262	102	89	93	148	−0.791	2.1E-04	0.001
zma-miR166a-5p	160	136	174	124	59	83	61	72	−0.895	2.7E-04	0.002
zma-miR5139	180	152	302	266	107	89	95	156	−0.759	3.5E-04	0.002
zma-miR156a-i, l-5p	1487	1671	1632	1672	1491	1922	1808	1658	0.327	4.3E-04	0.002
zma-miR390a, b-3p	31	32	31	50	16	14	11	8	−1.27	0.002	0.008
zma-miR166c-5p, e	187	142	142	213	86	111	93	61	−0.721	0.002	0.009
zma-miR169c-3p	142	101	156	181	66	106	57	67	−0.750	0.002	0.010
zma-miR396a, b-3p	112	114	108	78	30	71	46	61	−0.797	0.003	0.011
zma-miR827-3p	319	320	274	333	134	220	165	217	−0.549	0.003	0.012
zma-miR169f-5p, g, h	31	28	35	57	18	14	13	11	−1.13	0.004	0.015
zma-miR167c-3p	4	13	3	0	0	0	0	0	−3.99	0.005	0.018
zma-miR156h-3p	58	79	73	60	23	40	38	31	−0.800	0.006	0.021
zma-miR399a, c, h-3p	34	32	17	18	5	9	8	17	−1.240	0.007	0.026
zma-miR156i-3p	9	0	3	4	0	0	0	0	−3.77	0.008	0.028
zma-miR156a-3p	11	9	3	4	16	31	17	8	1.45	0.009	0.030
zma-miR167e-3p	29	16	31	32	55	46	25	42	0.837	0.010	0.032
zma-miR167a-d-5p	54	47	45	57	57	86	65	72	0.670	0.014	0.045
zma-miR398b-5p	58	73	69	106	25	31	59	42	−0.670	0.015	0.048

miRNAs that were differentially regulated by mild water-deficit stress (FDR <0.05) are listed first followed by miRNAs differentially regulated by severe water-deficit stress (FDR <0.05). The miRNA abundance in each library is represented in RPTM. WWR2-WWR5 are libraries constructed from primary root growth zone RNA under well-watered conditions, 3R1-3R4 from mild water deficit-stressed, and 16R1-16R4 severe mild deficit-stressed treatments. The log_2_FC, *p*-value, and false discovery rate (FDR) were calculated with EdgeR

To verify the results obtained by small RNA-seq, the relative abundances of 11 miRNAs in the primary root growth zone were measured by stem loop SLRT-qPCR (Additional file 11: Figure S3). The SLRT-qPCR data demonstrate a relatively strong correlation between the transcript expression values and trends in accumulation and depletion between the two methods of analysis for both the mild (Spearman’s correlation coefficient - rho = 0.83, *p* = 0.042) and severe water deficit treatments (Spearman’s correlation coefficient - rho = 0.89, *p* = 0.0068). The correlations are not perfect which may be a reflection of the relative sensitivities of the two methods to determine transcript abundance. The data also must be viewed with the following caveat. While the stem-loop method for miRNA quantification is specific enough to distinguish between single base differences between miRNAs [39], SLRT-qPCR is still not specific enough to distinguish between all individual miRNA family members or between a miRNA and isomiRs with a 5′ and/or 3′ nucleotide extension. In these cases the results may then represent the average change in the abundance of multiple miRNAs and/or isomiRs that are regulated differently under water deficit stress.

### Identification of gene transcripts regulated by primary root growth zone-expressed miRNA and changes in abundance under water deficit stress

To experimentally identify candidate transcripts regulated by miRNA cleavage in the primary root growth zone, degradome libraries were constructed from a single biological replicate for each water stress condition (Additional file 6: Table S6). The target transcripts of known maize miRNAs annotated in miRBase were predicted based on the expected cleavage site with in the miRNA complementary region as identified in the degradome sequence data. In total 223 different miRNA-regulated gene transcripts were detected by degradome sequencing of primary root growth zone tissue grown under well-watered, mild, or severe water deficit conditions (Additional file 7: Table S7). One hundred twenty-four of the miRNA-regulated gene transcripts were predicted to be targets of primary root growth zone-expressed miRNAs identified in this study by small RNA-seq (Additional file 7: Table S7). The transcripts were predicted to be the targets of 37 different miRNAs belonging to 22 of the 26 miRNA families that were detected in the primary root growth zone. The primary root growth zone-expressed miRNA family for which no target transcripts were detected were miR2118, miR5139, miR8155, and miR8175. Targets of additional miRNA families that were not detected in the primary root growth zone by small RNA-seq in this study were also detected. These were miR162, miR172, miR482, and miR2275. Roughly 30% of the miRNA-target transcript interactions that were identified were conserved (Additional file 7: Table S7) when being compared to similar relationships between the miRNA and transcript targets in Arabidopsis and rice [40] .

**Fig. 3:**
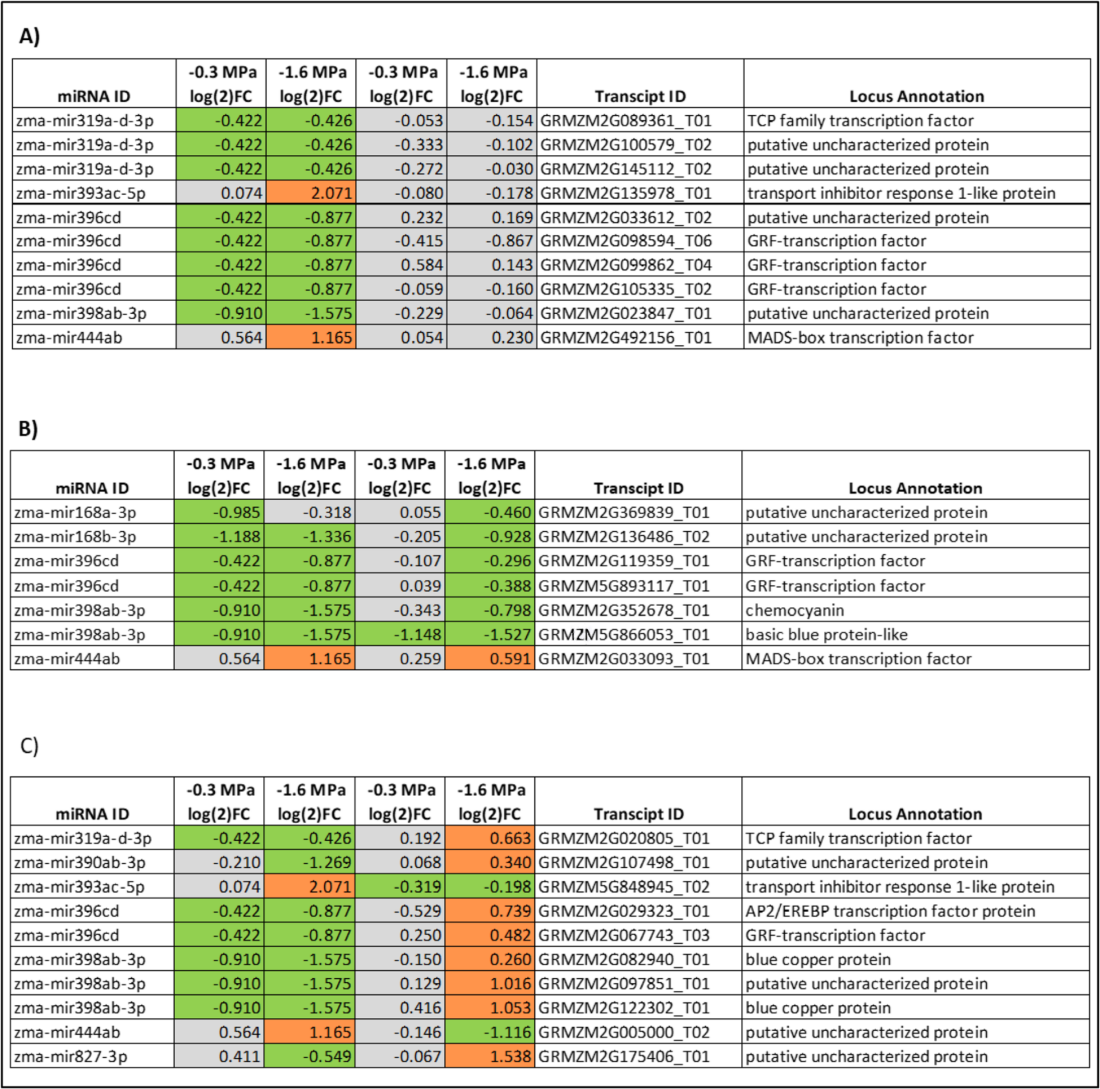
The abundance of water deficit-responsive miRNAs and the abundance of the target transcripts they regulate under mild and severe water deficit stress. Abundance is presented as the log [2] FC in transcript abundance under water deficit stress relative to well-watered conditions. Red indicates a significant increase in target transcript or miRNA abundance under water deficit stress, green indicates a significant decrease in abundance (FDR < 0.05). Gray values were not statistically significant. **a** The predicted target transcript of the water deficit-responsive miRNA was not differentially regulated under any water deficit stress. **b** Water deficit-induced regulation of predicted regulatory miRNA is positively correlated with the regulation of the target transcript. **c** Water deficit-induced regulation of predicted regulatory miRNA is inversely correlated with the regulation of the target transcript

To assess whether the abundance of miRNA-regulated gene transcripts detected by degradome sequencing changed under water deficit conditions, transcriptome sequencing was performed on primary root growth zone tissue grown under identical conditions. Twenty-seven gene transcripts predicted to be regulatory targets of water deficit-responsive miRNAs were detected by RNA-seq (Additional file 8: Table S8). The abundance of 10 of the gene transcripts predicted to be regulated by a water deficit responsive miRNA did not change significantly under water deficit (Fig. 3a). However, seventeen of the 27 gene transcripts were differentially regulated (FDR <0.05) under at least one water deficit condition (Fig. 3b,c). Seven of these were part of a miRNA-transcript pair in which the change in the abundance of the miRNA was positively correlated with the change in the abundance of the transcript (Fig. 3b). And 10 were part of a miRNA-transcript pairs in which an increase or decrease in miRNA abundance was coordinated with an inverse change in the abundance of the target transcript (Fig. 3c). The inverse relationship in the abundance of miRNA to transcript abundance observed for these 10 miRNA-transcript pairs is suggestive of a regulatory relationship between the miRNA and predicted target transcript. However, further experimental validations is required to confirm or eliminate whether any of the transcripts identified in this study are direct regulatory targets of water deficit stress-induced miRNAs.

### Identification of a water deficit-induced miR399 target mimic

**Fig. 4:**
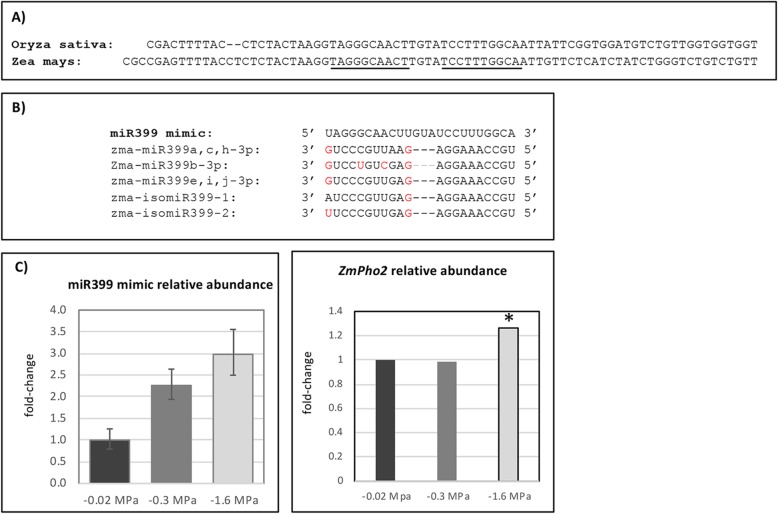
**a** Alignment of DNA sequences of predicted *Oryza sativa* miR399 target mimic (BU673244) identified in Franco-Zorrilla et al. (2017) surrounding the region complementary to miR399 and the predicted *Z. mays* target mimic. Underlined nucleotides are complementary to *Zea mays* miR399-3p family members. **b** Alignment of the miR399-complementary region of the target with miR399-3p and miR399 isomiRs expressed in the primary root growth zone. Red letters for the miR399 and miR399 isomiR nucleotides are not complementary to the miR399 target mimic. **c** Mean relative transcript abundance and standard error of miR399 target mimic in the PRGZ under different water deficit conditions measured by RT-qPCR. **d** Mean relative transcript abundance of pho2 in the PRGZ under different water deficit conditions measured by RNA-seq. The asterisk (*) denotes that pho2 abundance was significantly different under moderate stress

In these experiments, miR399e, i, j-3p was among the most strongly regulated miRNAs under both mild and severe stress. However, none of the reported targets of miR399e, i, j-3p were identified by degradome sequencing. Targets of the miRNA derived from the 5′ arm of the miR399 precursor, miR399e, i, j-5p, were identified and included two ubiquitin conjugating enzymes, but rather than being inversely correlated with either miR399e, i, j-3p or -5p abundance these ubiquitin conjugating enzyme transcripts were also more abundant under water deficit stress. miR399 has been well characterized as a mobile signal communicating Pi status from shoot to root and regulating phosphate uptake through its target ubiquitin conjugating enzyme, pho2 [35–37]. Another conserved component of the miR399-pho2 signaling pathway that has a role in communicating shoot Pi status to the root is a miRNA target mimic [41–43]. To determine whether a miR399 target mimic that could interrupt the cleavage of target transcripts existed in maize, the sequence of the rice miR399 target mimic was Blast-searched against the maize EST libraries. A contig was assembled from these ESTs, and the contig was aligned to the B73 RefGen_v3 genome assembly. An 82-nucleotide region with high sequence similarity to previously identified miR399 target mimics was located on chromosome 1 of the maize genome (Fig. 4a). The sequence included a region that is nearly identical to multiple miR399 family members (Fig. 4b). Transcription of this RNA was confirmed by RT-qPCR. Furthermore, RT-qPCR analysis showed that the RNA transcript was more abundant in the growth zone of water deficit-stressed compared to well-watered roots in well-watered roots (Fig. 4c).

## Discussion

Root growth adaptation to soil water deficit conditions is under the control of complex gene regulatory networks [5, 19, 44]. Mapping gene regulatory networks involves identifying the components (regulatory factors and cis-elements) and investigating the interactions among molecules under strictly controlled, reproducible experimental conditions. The vermiculite culture system used in this study is an established system that has been used extensively to apply defined water deficit conditions to the maize seedling primary root, and is effective for reducing confounding factors inherent in administering water deficit stresses [5, 14, 25]. In this framework, profiling the patterns of differentially expressed regulatory molecules within the primary root growth zone is a powerful approach for mapping the regulatory networks that control root growth adaptation to soil water deficit conditions to identify prospective targets for favorably manipulating plant drought tolerance.

Among different classes of regulatory molecules, miRNAs have been implicated as key post-transcriptional regulators of root system architecture and response to water deficit stress [37]. Using small RNA-seq we detected the presence of 79 miRNAs and multiple isomiRs belonging to 25 miRNA families in the maize seedling primary root growth zone under different water status regimes (vermiculite Ψ_w_ of − 0.02 MPa, − 0.3 MPa, or − 1.6 MPa) (Additional file 4: Table S4 and Additional file 5: Table S5). Abundant miRNA families detected in the 1-cm apical primary root growth zone were hypothesized to have roles in regulating maize seedling primary root growth and development. This is consistent with prior studies which have reported that several of these families (including miR156, miR159, miR160, miR165/miR166, miR167, miR319, miR390, miR393, and miR827) modulate different facets of root system architecture [37, 45]. The most abundant miRNA family detected in the primary root growth zone, miR166, is also well-characterized as a key regulator of primary root growth and development. miR166 regulates both root vascular patterning [36, 37] and root apical meristem (RAM) size [36, 37]. Expression of miR166 in the 1-cm apical primary root growth zone which includes the RAM is likely at least in part maintaining root apical meristem activity and root growth by restricting the expression of a group Class III HD-Zip transcription factors [37, 38]. In these experiments, water deficit-responsive miR166 family members were significantly less abundant in the primary root growth zone of water deficit-stressed seedlings relative to non-stressed seedlings which could contribute to the decrease in the rate of primary root growth that is observed in water deficit stress [25].

A large number of miRNAs that were detected in the primary root growth zone contained sequence variations relative to the canonical sequence annotated in miRBase and were considered to be isomiRs. IsomiRs originate from the canonical miRNA locus [20, 31], and length and/or sequence variants are generated during miRNA biogenesis by imprecise or alternative cleavage during pre-miRNA processing or by post-transcriptional RNA modifications [20, 31]. IsomiRs are categorized as 5′, 3′, or internal miRNA variants based on the location of the miRNA sequence polymorphism. The isomiRs detected in this study included 5′ and 3′ miRNA variants that were truncated, extended, or polymorphic at either or both ends, and internal miRNA variants with internal polymorphisms (in comparison to the canonical miRNA sequence). The miRNA sequence polymorphisms have the capacity to alter target specificity, miRNA stability, and/or localization [20]. However, the extent to which isomiRs actually do alter or increase the regulatory repertoire of a single precursor sequence is not clear [31]. Differentially-regulated isomiRs have been identified in response to temperature stresses and under phosphorous deficiency [31, 46, 47]. The isomiRs detected in this study present the potential to increase the complexity of miRNA-mediated regulatory networks in the primary root growth zone. However, their authenticity and the identity of their targets need experimental validation to contribute to the growing body of evidence for isomiR regulation of plant responses to abiotic stresses, and to ascertain if these isomiRs are physiologically significant.

Growth of the maize root system is less inhibited by water deficits relative to growth of the shoot [5, 11, 12, 14]. In the present study, shoot growth was inhibited by approximately 70% under the mild stress treatment and was virtually arrested under the severe stress treatment compared with well-watered control seedlings [5, 15]. Elongation of the primary root is also inhibited under these water deficit conditions, but less so than shoot growth, and substantial root growth continues at a Ψ_w_ of − 1.6 MPa [5, 14, 25]. It is known from transcriptomic and large-scale transcript profiling studies that the networks of genes that are perturbed in the primary root growth zone under water deficit stress are extensive and distinctive depending on the degree and duration of the stress exposure [5, 18, 44]. The two stresses that were imposed in this study, − 0.3 MPa (mild) or − 1.6 MPa (severe), were selected to uncover different miRNA-mediated regulatory networks that underlie primary root growth zone responses to different water deficit levels [5]. Using genome-wide small RNA-seq to measure relative transcript abundance, 34 water deficit stress-responsive miRNAs belonging to 17 miRNA families were identified in the primary root growth zone. Previously, Aravind et al. [24] identified 13 “drought-related” miRNA families with members that responded to a water deficit treatment in maize seedling leaf tissue. In both studies, miRNAs belonging to the miR156, miR159, miR166, miR169, miR390, miR393, miR396, and miR399 were responsive to water deficits. However, Aravind et al., did not find that the miR167, miR168, miR319, miR398, miR408, or miR444 miRNA families were responsive to treatments as found in this study. These differences could be a product of the different treatment conditions that were applied in the two studies, or could reflect developmental or tissue specific regulatory roles. It appears that Aravind et al. [24] quantified miRNA abundance in whole maize seedling leaves whereas this study measured miRNA abundance only in the maize seedling root growth zone. It is possible that these miRNA families have specific roles in regulating root growth under water deficit stress. Several of the water deficit-responsive miRNA families identified in this study, including miR166, miR167, miR168, miR319, miR393, and miR408, have also been characterized as drought-responsive in other plant species. Thirty-one water deficit responsive miRNAs were differentially regulated under severe water deficit conditions, and 10 were differentially regulated under mild water deficit stress (Table 1). Although it is difficult to define what level of abundance constitutes a physiologically relevant amount of an miRNA, it seems likely that at least three of the miRNAs that were classified as significantly differentially regulated, zma-miR169r-3p in the − 0.3 MPa analysis and zma-miR167c-3p and zma-miR156i-3p in the − 1.6 MPa analysis, are not sufficiently expressed to deliver a biological signal. Likewise it is also difficuly to assess what constitutes a biologically relevant magnitude for a fold change in an miRNA in response to an environmental stimulus. The miRNAs listed in Table 1 all exhibit a significant change in abundance in response to a water deficit, however zma-miR156 a-I,1-5p, zma-miR167 a-d, 5p and zma-miR167 e-3p have only a modest change in abundance which may indicate that their role in the response is minimal. The larger number of differentially regulated miRNAs under severe stress than under mild stress closely mirrors the results of a previous study by Seeve et al. [5] which examined differential regulation of TF transcripts in the primary root growth zone under the same stress conditions. Although there were far fewer miRNAs that were differentially regulated by mild water deficit stress, these miRNAs are of interest for their potential roles in maintaining adaptive root elongation rate at less severe soil water deficits or early on in the progression of a drought before the soil water deficit becomes severe. The miRNA families differentially regulated by mild water deficit stress were miR159, miR167, miR168, miR169, miR319, miR396, miR399, and miR408 (Fig. 2).

The miRNA-regulated targets identified in the primary root growth zone possessed a wide range of functions including a large number of TF gene transcripts. Several of the miRNA families each regulated multiple members of a single TF gene family (Additional file 6: Table S6). Several of these miRNA-TF modules are widely conserved and have been described in other species including the miR156-squamosa promoter-binding protein [48, 49], miR159-MYB TF [48, 49], miR160-auxin response factor (ARF) [48, 50], miR166-HD-Zip [38], miR169-NF-YA [48, 51] miR171-GRAS TF [48], miR319-TCP family TFs [52], miR396-GRF TFs [52]. Further, a large number of the gene transcripts identified as targets of water deficit-sensitive miRNAs in the degradome libraries were transcription factors (Additional file 7: Table S7). Sunkar et al., [53] observed that nearly all miRNAs that regulate TF gene transcripts are stress-responsive. These miRNA-TF regulatory modules can have a profound effect on plant stress physiology by regulating downstream signal transduction [53] and are thus attractive targets for improving drought tolerance.

The miRNA target transcripts detected by degradome sequencing are predicted to be gene targets regulated by miRNA-guided RNA degradation. In the miRNA:target transcript regulatory relationship, an increase in miRNA abundance under water deficit stress is accompanied by a decrease in the abundance of the full-length transcript, and vice versa. Ten of 27 water deficit-responsive miRNA-transcript modules identified in this study fit this description, whereas the remaining miRNA-transcript modules exhibited incoherent regulation (parallel changes in miRNA and target abundance) [54] or unchanged target abundance. Multiple studies have demonstrated that the characteristics of the relative expression patterns of the miRNA and its target transcript are shaped by the larger regulatory network in which the miRNA-transcript module is involved [43, 54, 55]. For example, independent transcriptional regulation of the miRNA and its target, or feedforward and feedback loops involving miRNA-TF modules may obscure the inverse miRNA-target relationship [43, 54, 55]. These regulatory mechanism have important roles in buffering target transcript fluctuations, or in establishing expression boundaries and abundance thresholds [55]. In addition to miRNA-mediated regulation of gene transcripts, reciprocal transcript regulation of miRNA activity can conceal the inverse miRNA-target relationship [43]. As illustrated in the results of the degradome analyses, typically multiple target transcripts are regulated by a single miRNA. Depending on certain transcript characteristic and the relative rates at which the various target are transcribed, through competition some miRNA transcripts can substantially diminish the activity of miRNAs on other targets without a notable effect on the abundance of the transcript itself [43]. In addition to the regulatory network dynamics in which the miRNA-transcript module is involved, spatial separation of the miRNA and the target transcript could explain incoherent or unchanged miRNA target abundance. As the measurements made in this study represent snapshots of the average abundance of miRNAs and transcripts in a population of cells, these results do not reflect the events occurring at the cellular level. Thus, experimental validations is required to confirm or eliminate any of the 27 transcripts identified in this study as direct regulatory targets of water deficit stress-induced miRNAs.

The first example of an endogenous transcript affecting the regulatory activity of a miRNA on other transcripts was regulation of miR399 activity by the non-coding RNA IPS1 (induced by phosphate starvation) in *A. thaliana* [41]. IPS1 includes a motif with near-perfect complementarity to miR399, but with a mismatch at the miRNA cleavage site that prevented transcript cleavage. Under phosphorous-deprivation, miR399 indirectly regulates the expression of multiple phosphate transporters and a protein involved in loading inorganic phosphate into the xylem through its target transcript, a ubiquitin conjugating enzyme, PHO2, to maintain phosphate homeostasis throughout the plant [41, 42]. Levels of the cleavage-resistant IPS1 transcript also rise under phosphorous limited conditions [41, 42] sequestering miR399 and reducing its activity on PHO2 and other targets, a regulatory mechanism coined by Franco-Zorrilla et al. [41] as “target mimicry”.

Recently, Du et al. [56] demonstrated that conservation of a Pi-deficiency induced regulatory module involving miR399b, *ZmPho2* (GRMZM2G381709), and miRNA target mimic extends to maize as well. They independently identified the same miR399 target mimic that was identified in this study, naming it *pilncr1*. In this regulatory module, *pilncr1* inhibits miR399-guided cleavage of *ZmPho2* [56]. We were unable to design primers specific to ZmPho2 to measure transcript abundance by RT-qPCR, but did find by RNA-seq that the abundance of ZmPho2 was significantly higher in the PRGZ under severe stress (Fig. 4d).

Although generally regarded as a regulator of nutrient deficiency responses [53], particularly phosphorous deficiency, drought and salt-regulated changes in miR399 transcript abundance has been reported in previous studies [21]. However, its role in abiotic stress tolerance has not been characterized. To our knowledge, this is the first time that an increase in the abundance of a putative miR399 target mimic under water deficit stress has been reported. Uptake of mineral nutrients including phosphorous is reduced under soil water deficits. However, it is expected that at this early stage in seedling development the seed provides adequate nutrient resources to the developing seedling suggesting that the differential regulation of the miR399 family members is responding specifically to the effects of the water deficit stress induced in the primary root growth zone. Further studies evaluating the nutrient status of water deficit stressed seedlings at this stage of development are required to confirm this hypothesis. Plant physiological adaptations such as modifications to the root system architecture to enhance soil exploration and absorption, adjustments of the root:shoot ratio are common to both water deficit and low phosphorous stresses [57]. The involvement of the miR399-*pilncr1* module in overlapping regulatory networks governing plant responses to both phosphorous deficiency and water deficit stress remains an area of focus for future research.

## Conclusion

In summary, of the 79 miRNAs that are expressed in the primary root growth zone of maize seedlings, 34 miRNAs are regulated by either mild (− 0.3 MPa) or severe (− 1.6 MPa) water deficit stress. In addition, 223 miRNA-regulated gene transcripts in the primary root growth zone were identified by degradome sequencing. Only a small fraction of the miRNA-transcript pairs were inversely regulated under water deficit stress. We identified a water deficit-responsive miR399 target mimic and its putative interaction with miR399 family members. Whether this target mimic is regulating root growth responses to water deficit stress through the conserved phosphate-deficient responsive regulatory pathway is unknown. However, we do not hypothesize that the differential regulation of the miR399 target mimic was in response to nutrient deficiencies that may result as a by-product of water deficit stresses.

## Methods

### Plant material and water deficit treatments in a vermiculite system

Seeds of maize line B73 were harvested from field-grown plants planted at the University of Missouri Genetics Farm. The original B73 line was obtained from Dr. Sherry Flint-Garcia, USDA-ARS Plant Genetics Unit, Columbia Missouri. Water deficit treatments were applied in a non-transpiring vermiculite culture system developed by Sharp et al. [14] and which has been further described in Seeve et al. [5]. Briefly, surface sterilized maize seeds were imbibed for 24 h at room temperature in aerated 1 mM CaSO4 solution. Imbibed seeds were planted in boxes of vermiculite hydrated to the drip point with 1 mM CaSO_4_, and germinated in the dark at 29 ± 1 °C and near-saturation humidity until primary roots had reached 15–25 mm in length. Seedlings were transplanted to Plexiglas boxes containing vermiculite moistened with 1 mM CaSO4 to achieve water potentials of Ψ_w_ of − 0.02 MPa, (well-watered), Ψ_w_ = − 0.3 MPa (mild water deficit stress), or Ψ_w_ = − 1.6 MPa (severe water deficit stress), and grown in the dark at 29 °C and near saturation humidity [5, 14, 58]. At 26 h after transplanting the primary root growth zone, consisting of the apical 1 cm of the primary root and including the root cap, was harvested from each seedling. In the water-deficit treatments in this system, seedling water status decreases gradually after transplanting as tissues lose water to the medium. It was previously shown that the root tip Ψ_w_ declines to near-stable levels by 26 h after transplanting in both the mild and severe water deficit treatments [5]. The sampled region encompassed the majority or entirety of the root growth zone, depending on the treatment. The growth zone in the maize primary root has been shown to extend approximately 12 mm from the apex under well-watered conditions and to progressively shorten with increasing water deficit stress [14, 59]. Five root segments were pooled to represent a single biological replicate. Collected tissues were immediately frozen in liquid N_2_. Water potentials of the vermiculite (prior to transplanting and at harvest) and the root growth zone (at harvest) were determined by isopiestic thermocouple psychrometry [60].

### Small RNA library preparation, sequencing, and analysis

Six biological replicates for each of the three treatments, well-watered, mild water deficit, and severe water deficit, were used for small RNA sequencing. Each biological sample was ground to a fine powder in liquid nitrogen, and small RNAs were extracted using the mirPremier microRNA Isolation kit (Sigma-Aldrich, St Louis, MO) according to the manufacturer’s instructions. Small RNA library preparation and sequencing was performed by Q2 Solutions Expression Analysis, LLC (Morrisville, NC). The small RNA libraries were analyzed as described previously [40, 61, 62]. Briefly, raw data was processed to trim adapter sequences and to filter low quality reads that have low scoring nucleotides (< 25) in the first 25 nucleotides of the raw read. Short reads of less than 18 nucleotides were removed and a count was created for each unique sequence (after removing redundant reads).

The maize genome was downloaded from Gramene (http://ftp.gramene.org/archives/PAST_RELEASES/release41/data/fasta/zea_mays/dna/, v3.22). The cDNA of maize were downloaded from the Maize Genetics and Genomics Database (http://ftp.maizegdb.org/MaizeGDB/FTP/GeneModels_5b+/, version 3.22) [63]. Unique sequences were mapped in parallel to the genome, mature mRNAs of maize from iRBase (r21), non-coding RNA databases, including Rfam (r11) [64], NONCODE (v3.0) [65], GtRNAdb [66], and repeat databases, including Plant Repeat Databases [67] and Repbase (r20) [68] using SOAP2 [69]. Unique sequences were mapped to the mature miRNAs of maize and mature miRNAs of other plant species in miRBase (r21) [70] to identify mature miRNAs. Annotated maized miRNAs were named according to their record in the miRBase and miRNAs with mapped sequence reads were named as miR followed by the family number and a character in alphabetic order. All mature miRNAs in maize and mature miRNAs reported in other plants were used in these analyses. Normalized reads per 10 million tags (RPTM) were calculated for each miRNA.

Only miRNAs with greater than or equal to 1 RPTM in at least 3 of the 4 libraries in a treatment group were retained for statistical analyses described here. The non-normalized frequencies of mature miRNAs were entered in the R package edgeR [71] to examine sample relationships and to identify differentially regulated miRNAs. The similarity of samples within each treatment was examined by using the edgeR MDS plot function (Additional file 9: Figure S1). Suspect samples were identified and removed as described in detail in Additional file 9: Figure S1 which reduced the number of biological replicates per treatment to four. Raw library counts were trimmed by the mean of M-values (TMM) and normalized. The TMM-normalized data were fitted to negative binomial general linear models. Three pairwise comparisons were made comparing miRNA abundance in each water deficit treatment with the control, and comparing miRNA abundance in the mild and severe water deficit treatments. The quasi-likelihood F-test was applied to test for differential expression. Resulting *P*-values were corrected for multiple testing according to Benjamini & Hochberg [72]. Tests with FDR < 0.05 were considered significant.

### Measurement of miRNA relative abundances by stem-loop RT-PCR

The relative abundances of 11 miRNAs that were differentially regulated under water deficit conditions (FDR < 0.05) were measured by stem-loop RT-PCR (SLRT-qPCR). The RNA Stem-loop RT primers, and qPCR forward and reverse primers were designed according to Chen et al. [39] and are listed in (Additional file 1: Table S1). Reverse transcription and quantitative PCR reactions were carried out as described in Liu, Kamari, Zhang, Zheng & Ware et al. [73]. Briefly, quantitative PCR reactions were performed in triplicate on a Rotor-Gene 6000 with SYBR Green PCR master mix (Qiagen, Carlsbad, CA) for detection. The amplification of single products was confirmed by dissociation curve analysis. As recommended by Meyer, Pfaffl, & Ulbrich [74] miRNA relative quantification was calculated by normalizing miRNAs to an invariant reference miRNA. Two different miRNAs were determined to be stably expressed across water deficit treatments, miR168a and miR169c, and were used as reference miRNAs to calculate miRNA relative abundance by the ddCt method [75].

### Degradome library preparation, sequencing, and analysis

Maize root tips were collected as previously described and approximately 45 μg of total RNA was extracted from the root tips using the TRIzol method of extraction. Total RNA was treated with Turbo DNase (Life Technologies, Waltham, MA) to remove all contaminating genomic DNA and cleaned using the Zymo RNA Clean & Concentrate kit (Zymo Research, Irvine, CA) to remove contaminants. RNA quality was assessed by micro-capillary based electrophoresis (**Agilent Bioanalyzer 210: Agilent, Santa Clara, CA)**. Poly-A RNA was isolated from approximately 30 μg total RNA using the dynabeads mRNA DIRECT purification kit (**Life Technologies Corporation**, Carlsbad, CA). Degradome libraries were prepared according to Willmann, Berkowitz, & Gregory [76] with the modification that libraries were prepared using components from the NEBNext Small RNA library preparation kit (New England BioLabs Inc. Ipswich, MA). A specially designed random hexamer primer, which includes the 3′ adapter sequence in its 5′ end, was used to randomly reverse transcribe RNA and incorporate the 3′ adapter sequence at the same time. The 3′ NEBNext adapter sequence is different from the TruSeq adapter sequence and so the random hexamer RT primer was modified to be compatible with the NEBNext kit: (5′- CTGGAGTTCAGACGTGTGCTCTTCCGATCTNNNNNN-3′). The cDNA libraries were PCR amplified for 15 cycles with indices to allow multiplexing during sequencing. Amplified libraries were analyzed by electrophoresis in a 1.5% agarose gel (Amresco LLC, Solon OH) and the region of the gel corresponding to approximately 150–600 bp in length was excised. Prior to sequencing, library concentrations were measured fluorometrically (Qubit, ThermoFisher, Waltham, MA) and fragment size distribution was determined by capillary electrophoresis using an ABI 3730xl DNA Analyzer (**Life Technologies Corporation**, Carlsbad, CA). Sequencing was performed using an Illumina HiSeq 2500 sequencing platform (Illumina, San Diego, CA). One degradome library was prepared for each treatment.

Degradome libraries were filtered to remove low quality reads that had low scoring nucleotides (< 25) in the first 20 nucleotides of the raw reads. Then, the first 20 nucleotides were removed from the raw reads. The counts of unique sequences were calculated after removing redundant reads. The unique sequences were mapped to the maize genome, maize cDNAs [63], the miRBase database (r21) [70], non-coding RNA databases, including Rfam (r11) [64], NONCODE (v3.0) [65], GtRNAdb [66], and repeat databases, including Plant Repeat Databases [67] and Repbase (r20) [68] using SOAP2 [69]. The target transcripts of known maize miRNAs annotated in miRBase were predicted using the SeqTar algorithm [62]. Conserved miRNA target transcripts with at least one valid read aligning to the 9th to11th positions of a miRNA binding site, and transcripts that aligned to the miRNA sequence with fewer than 4 mismatches were considered valid targets [62].

### Quantitative assessment of miRNA-regulated gene transcripts under water deficit stress by RNA-seq

Transcriptome sequencing was performed to assess the abundance of miRNA-regulated gene transcripts in the primary root growth zone of maize seedlings exposed to the three different water status regimes. Water deficit stresses and tissue collection were carried out in the same manner previously described. Sequencing was performed on three biological replicates consisting of five 1-cm primary root growth zone segments of maize seedling collected from one of the three treatments: well-watered (− 0.02 MPa), mild water-deficit (− 0.3 MPa), or severe water-deficit (− 1.6 MPa). Each biological sample was ground to a fine powder in liquid nitrogen. Total RNA was extracted using the RNEasy Plant Mini Kit (Qiagen, Carlsbad, CA) and treated with Turbo DNase (Life Technologies, Carlsbad, CA) to remove contaminating genomic DNA. Starting with 1 μg total RNA, RNA-Seq libraries were prepared using the NEBNext mRNA library prep kit for Illumina (New England BioLabs, Ipswich, MA). Sequencing was performed with the Illumina NextSeq 500 platform (Illumina, San Diego, CA). Following removal of adaptor sequences, quality control and read mapping were carried out using CLC Workbench (Version 6.8.1 Qiagen, Carlsbad, CA). Low quality nucleotides were trimmed and sequences with more than two ambiguous nucleotides and low-quality sequences (< 0.02) were filtered. From the remaining sequences, reads ≥36 nt were retained and mapped to the maize B73 genome and gene models (RefGen_v3 (Release 5b+)). Differential gene expression was analyzed using the R software package EdgeR as described previously [5].

### RNA isolation, cDNA synthesis, and RT-qPCR reactions

Water deficit-stress induced changes in the relative abundance of nine miRNA-regulated gene transcripts that were quantified with RNA-seq were validated with RT-qPCR. Total RNA was extracted from the primary root growth zone of five maize seedlings using the TRIzol method of extraction. Total RNA was treated with Turbo DNase (Life Technologies, Carlsbad, CA) to remove all contaminating genomic DNA, and cleaned up using the Zymo RNA Clean & Concentrate kit (Zymo Research, Irvine, CA) to remove contaminants. First-strand cDNA synthesis was performed on a 5-μg aliquot of the total RNA using SuperScript III (Invitrogen, Carlsbad, CA) and was primed with random hexamer primers (Invitrogen, Carlsbad, CA). Transcript abundance was measured by qPCR using SYBR® Green PCR master mix (Applied Biosystems, ThermoFisher, Waltham, MA) according to the manufacturer’s instructions. Quantitative PCR reactions were assayed in an ABI 7900HT detection system (**Life Technologies Corporation**, Carlsbad, CA) using the default fast cycling conditions (Stage 1: 50 °C for 2 min.; Stage 2: 95 °C for 10 min; Stage 3: 95 °C for 15 s, then 60 °C for 1 min; Repeat Stage 3, 40 cycles). Dissociation curve analysis was performed to confirm the selective amplification of a single transcript. The transcript abundance of each target was measured in every tissue in triplicate and in three biological replicates from each water-deficit treatment. The primers used in these reactions are listed in Additional file 2: Table S2.

## Supplementary information

**Additional file 1: Table S1.** List of miRNA SLRT-qPCR primers. The first column is the primer identifier and indicates the amplified miRNA (with the exception of the last primer which is the universal reverse primer). The second and third columns list the miRNA-specific forward primer sequence and the stem loop primer, respectively.

**Additional file 2: Table S2. **List of RT-qPCR primer sequences used to validate the relative abundances of miRNA-regulated gene transcripts measured by RNA-seq.

**Additional file 3: Table S3.** Summary statistics of results of high-throughput sequencing of small RNA libraries.

**Additional file 4: Table S4.** Counts of miRNAs detected in the primary root elongation zone under well-watered (WW), and mild and severe water deficit conditions. There are four biological replicates per water potential condition. miRNA abundances in each library are represented in RPTM (reads per ten million). Log fold-change calculations, *p*-values, and FDRs were generated with EdgeR. Significant tests (FDR < 0.05) are highlighted in bright yellow.

**Additional file 5: Table S5. **microRNA sequence variants (isomiRs) detected by small RNA-seq. The reference miRNA sequences are highlighted in gray. In the isomiR sequences, green indicates that the nucleotide in the isomiR is the next consecutive nucleotide in the miRNA stem loop sequence but is not in the mature miRNA reference sequence in miRBase, blue indicates that a nucleotide in the mature reference miRNA was not in the isomiR sequence, and red indicates a polymorphism in the isomiR sequence relative to the miRBase miRNA sequence.

**Additional file 6: Table S6. **Summary statistics of results of high-throughput sequencing of degradome libraries.

**Additional file 7: Table S7. **List of miRNA-regulated gene transcripts detected in the primary root growth zone by degradome sequencing, and EdgeR-calculated fold-change value. The predicted miRNA-target transcript interactions that are conserved in other species are listed in red. The fold-change value of significantly differentially regulated transcripts (FDR < 0.05) is highlighted with yellow. Transcripts that were not detected in the RNA-seq libraries are indicated with an #NA. **Treatment Library**—the library/libraries in which the target transcript was identified: WW, − 0.3, − 1.6 = A; WW, − 0.3 = B; WW, − 1.6 = C; − 0.3, − 1.6 = D; WW only = W; − 0.3 only = M; − 1.6 only = S; **miRNA ID**—the ID of the miRNA predicted to regulate the transcript; **Transcript ID**—the AGPv3 transcript identifier; **− 0.3 MPa/26 h**—abundance of the transcript in the mild water deficit treatment relative to its abundance in the well-watered treatment (log2FC); **− 1.6 MPa/26 h**—abundance of the transcript in the severe water deficit treatment relative to its abundance in the well-watered treatment (log2FC).

**Additional file 8: Table S8. **Abundance of selected miRNA-target mRNAs in the primary root elongation zone under well-watered (WW; − 0.02 MPa), and mild (MILD; − 0.3 MPa) and severe (MOD; − 1.6 MPa) water deficit conditions. There are three biological replicates per water potential condition. Transcript abundances in each library are represented in CPM (counts per million). Log fold-change calculations, *p*-values, and FDRs were generated with EdgeR

**Additional file 9: Figure S1. **A) Two-dimensional scatterplot of the biological coefficient of variation (BCV) among 18 biological replicates based on non-normalized counts of the 100 highest count miRNAs. The outlying samples that are indicated with * were removed. B) Two-dimensional scatterplot of the biological coefficient of variation (BCV) among 12 biological replicates (following removal of outliers) based on non-normalized counts of the 100 highest count miRNAs. In both A) and B) biological replicates grown in well-watered conditions are indicated with green, in mild water deficit stress with red, and in severe water deficit stress with black. Additional file 3: Figure S2: Plots of correlation between RNA-seq and stem-loop RT-qPCR results for the (A) -0.3 MPa vs WW, and (B) -1.6 MPa vs WW comparisons. The line of best fit is shown and the Spearman’s correlation coefficient (r) and p-values (p) are indicated on each plot

**Additional file 10: Figure S2.**The RPTM normalized values for all miRNA belonging to the same miRNA family in each sample were summed. High abundance (> 100,000 average RPTM per sample), moderate abundance (> 1000 to < 20,000 average RPTM per sample), and low abundance (< 1000 average RPTM per sample) miRNA families are grouped together

**Additional file 11: Figure S3.** Plots of correlation between RNA-seq and stem-loop RT-qPCR results for the (A) -0.3 MPa vs WW, and (B) -1.6 MPa vs WW comparisons. The line of best fit is shown and the Spearman’s correlation coefficient (r) and p-values (p) are indicated on each plot

## Abbreviations

miRNA: microRNA
RAM: root apical meristem
RT-qPCR: reverse transcription quantitative PCR
TF: transcription factor
