# Supplementary material for: Water-deficit responsive microRNAs in the primary root growth zone of maize
Source: BMC Plant Biol. 2019 Oct 24;19:447. doi: 10.1186/s12870-019-2037-y (PMC6814125; doi:10.1186/s12870-019-2037-y)

Figure S1. A) miRNA Library sizes after filtering out lowly expressed miRNAs. The left panel is library size before normalization. The right panel is library size after normalization. Libraries WWR6, 3R6, 16R6, and a lesser extent 3R5 are considerably smaller than the other libraries. This trend can also be observed in the sequencing summary statistics in Table S3. Sequencing depth of these four libraries is within the same range of the other libraries, but fewer reads in those libraries mapped to sequences in miRbase. This could suggest that the smaller miRNA library sizes for these four samples represent technical error introduced during small RNA library preparation. Smaller libraries are susceptible to RNA composition biases and may effect downstream differential gene expression analysis. **B)** miRNA abundances after filtering out lowly expressed miRNAs. A few highly expressed miRNAs make up a substantial proportion of the total library size. The abundance of zma-miR166-3p in libraries WWR6 and 3R6 is noticeably different from the other libraries. The difference in the abundance of zma-miR166-3p in these two libraries can effect on how frequently lower expressed miRNAs are sequenced, and as a result, can generate the false appearance that some other miRNAs are expressed at a higher level. **C)** MDS plot of sample relationships for all 6 replicates per treatment group. After filtering and normalization, the edgeR plotMDS function was used to examine the samples for outlier and other relationships. In the plot, samples within treatment groups were heterogenous and there was no clear separation among treatment groups. Distances among samples correspond to leading log-fold-differences between samples. We suspected that RNA composition biases among the libraries due to differences in library size and differences in the abundance of highly expressed miRNAs could be generating false log-fold differences and contribute to the sample heterogeneity observed in the plot. **D)** MDS plot of sample relationships after suspected outlier samples WWR6, 3R6, 16R6, and 3R5 were removed. To keep replicates equal within treatments, samples WWR1 and 16R5 were also removed, bringing the total to 4 replicates per treatment group. In this plot, some sample heterogeneity remained among samples within a treatment group, but treatment effect was observed in dimension 2. Samples belonging to each treatment group are separated on dimension 2.

A)
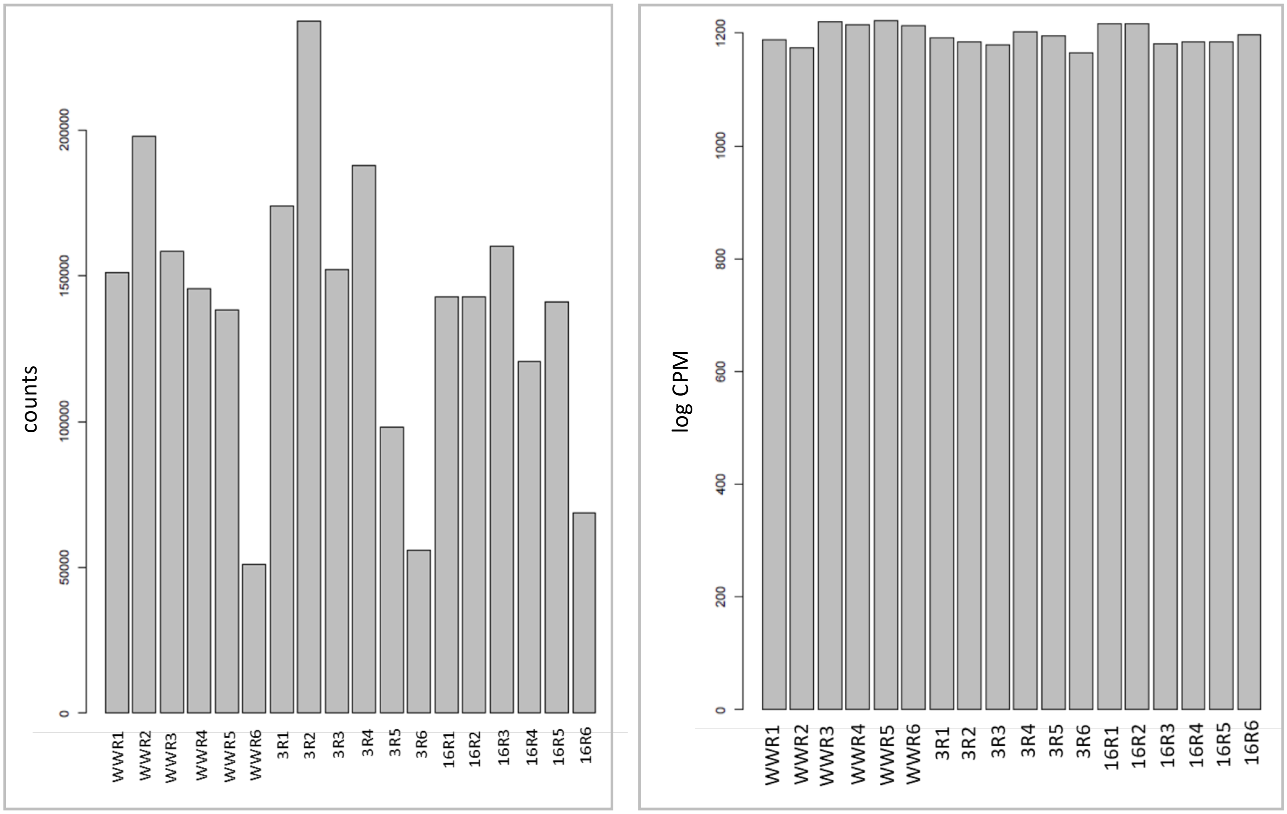
counts
0
50000
100000
150000
200000
WWR1
WWR2
WWR3
WWR4
WWR5
WWR6
3R1
3R2
3R3
3R4
3R5
3R6
16R1
16R2
16R3
16R4
16R5
16R6
log CPM
0
200
400
600
800
1000
1200
WWR1
WWR2
WWR3
WWR4
WWR5
WWR6
3R1
3R2
3R3
3R4
3R5
3R6
16R1
16R2
16R3
16R4
16R5
16R6

B)
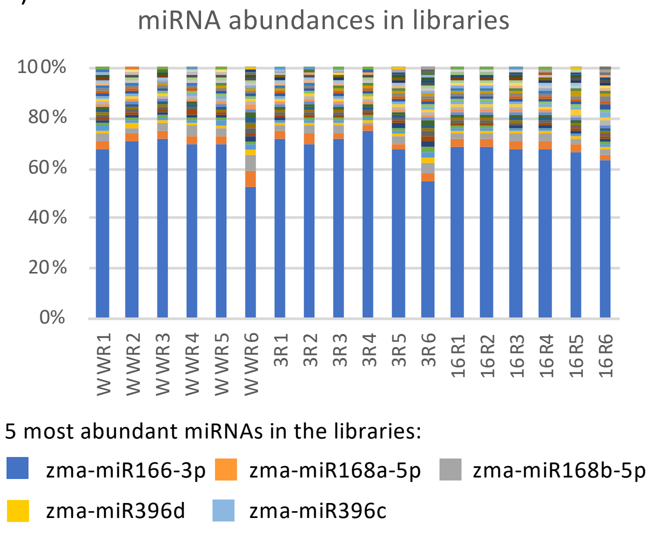
miRNA abundances in libraries
100%
80%
60%
40%
20%
0%
WWR1
WWR2
WWR3
WWR4
WWR5
WWR6
3R1
3R2
3R3
3R4
3R5
3R6
16R1
16R2
16R3
16R4
16R5
16R6
5 most abundant miRNAs in the libraries:
zma-miR166-3p
zma-miR168a-5p
zma-miR168b-5p
zma-miR396d
zma-miR396c

c)

MDS plot of sample relationships for all
6 replicates per treatment group

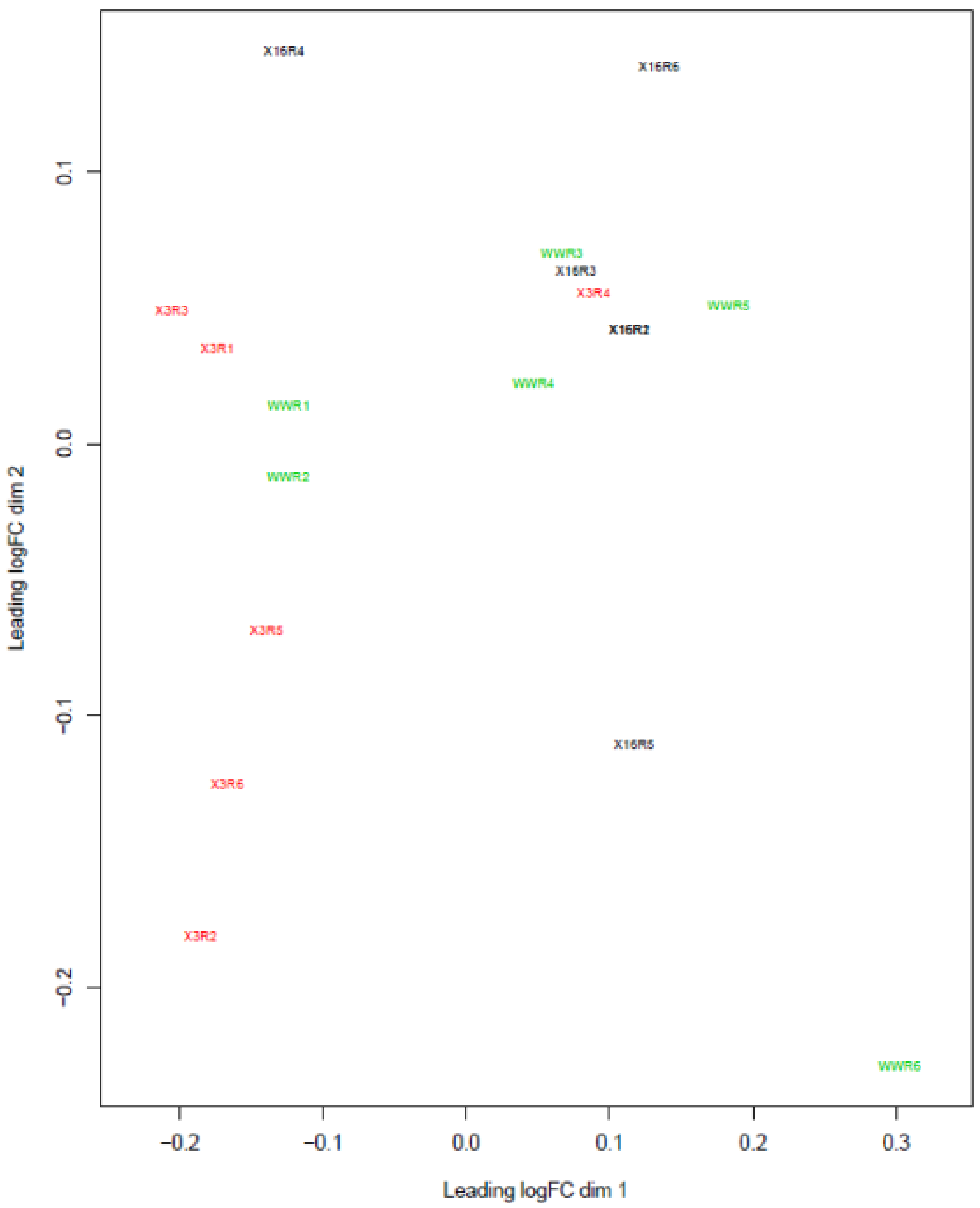

D)

MDS plot of sample relationships with suspected outlier samples removed (4 replicate samples per treatment)

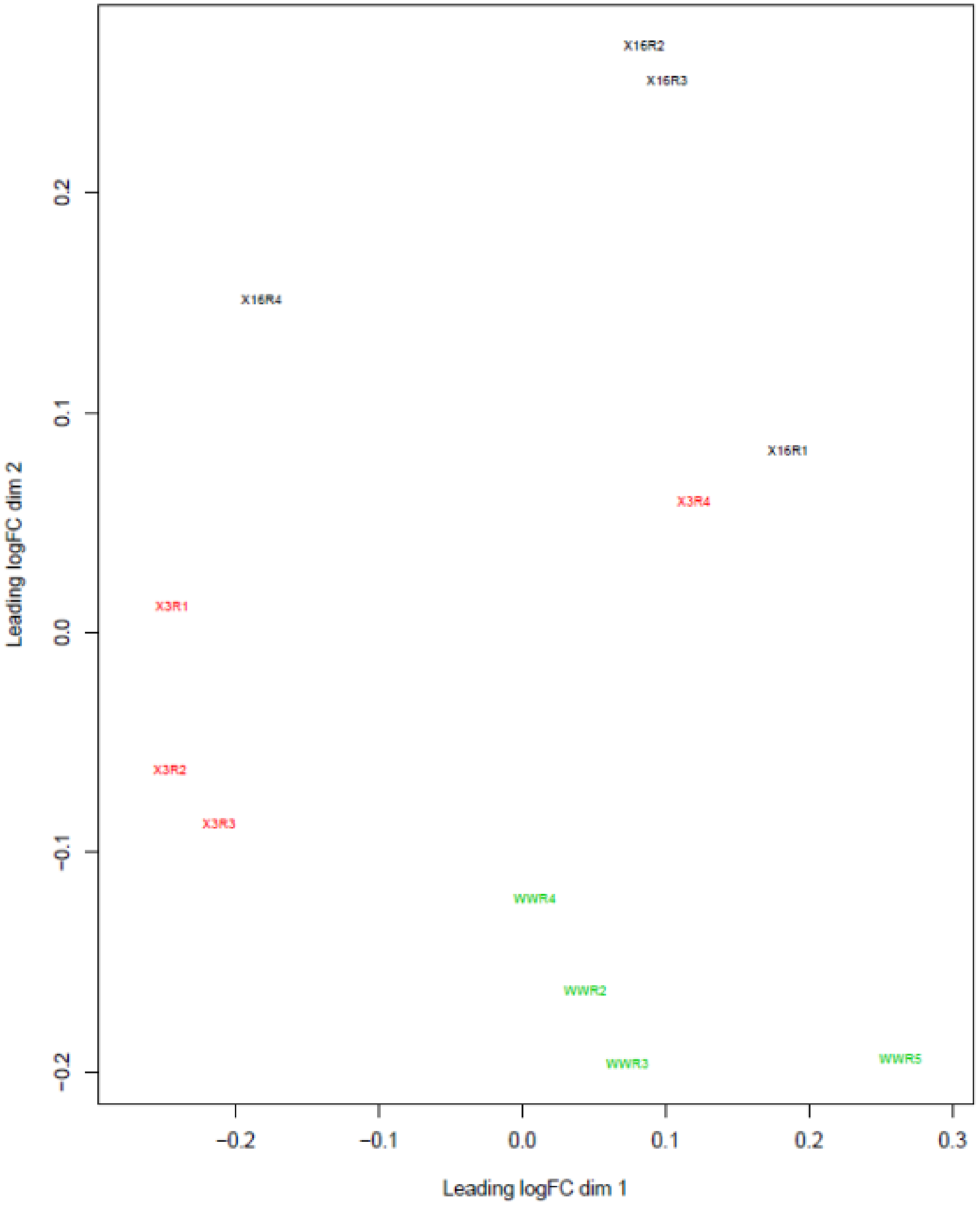

Supplement: Supplementary file 9 — Additional file 9: Figure S1. A) Two-dimensional scatterplot of the biological coefficient of variation (BCV) among 18 biological replicates based on non-normalized counts of the 100 highest count miRNAs. The outlying samples that are indicated with * were removed. B) Two-dimensional scatterplot of the biological coefficient of variation (BCV) among 12 biological replicates (following removal of outliers) based on non-normalized counts of the 100 highest count miRNAs. In both A) and B) biological replicates grown in well-watered conditions are indicated with green, in mild water deficit stress with red, and in severe water deficit stress with black. Additional file 3: Figure S2: Plots of correlation between RNA-seq and stem-loop RT-qPCR results for the (A) -0.3 MPa vs WW, and (B) -1.6 MPa vs WW comparisons. The line of best fit is shown and the Spearman’s correlation coefficient (r) and p-values (p) are indicated on each plot [file 12870_2019_2037_MOESM9_ESM.pdf]
