# Supplementary material for: Water-deficit responsive microRNAs in the primary root growth zone of maize
Source: BMC Plant Biol. 2019 Oct 24;19:447. doi: 10.1186/s12870-019-2037-y (PMC6814125; doi:10.1186/s12870-019-2037-y)

Figure S2. The RPTM normalized values for all miRNA belonging to the same miRNA family in each sample were summed. High abundance (> 100,000 average RPTM per sample), moderate abundance (>1,000 to <20,000 average RPTM per sample), and low abundance (< 1,000 average RPTM per sample) miRNA families are grouped together.

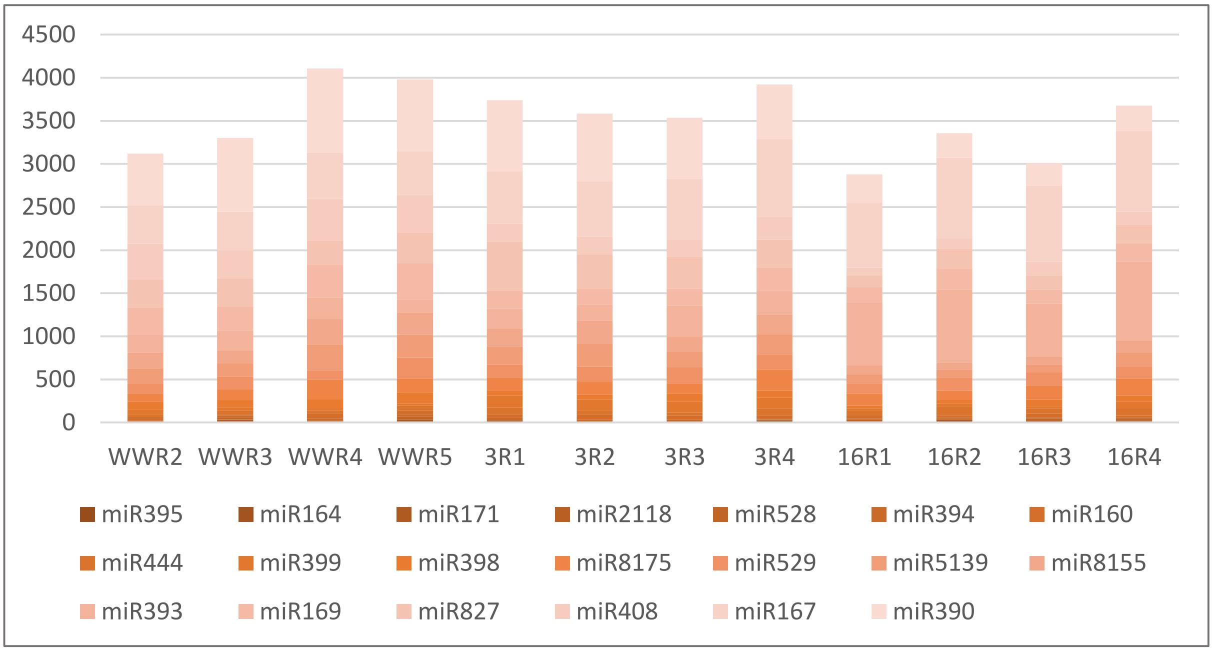

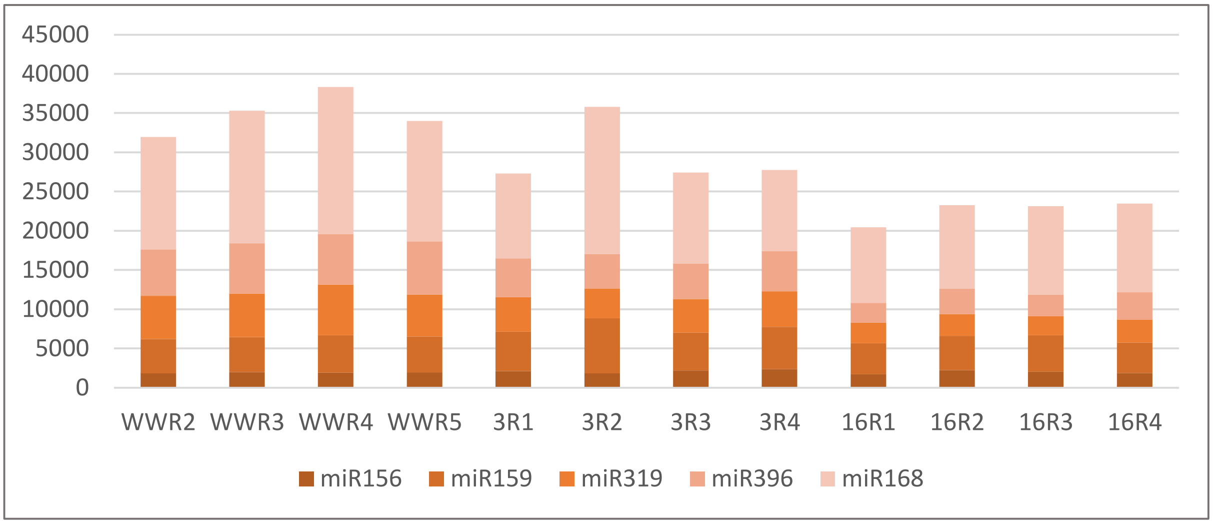

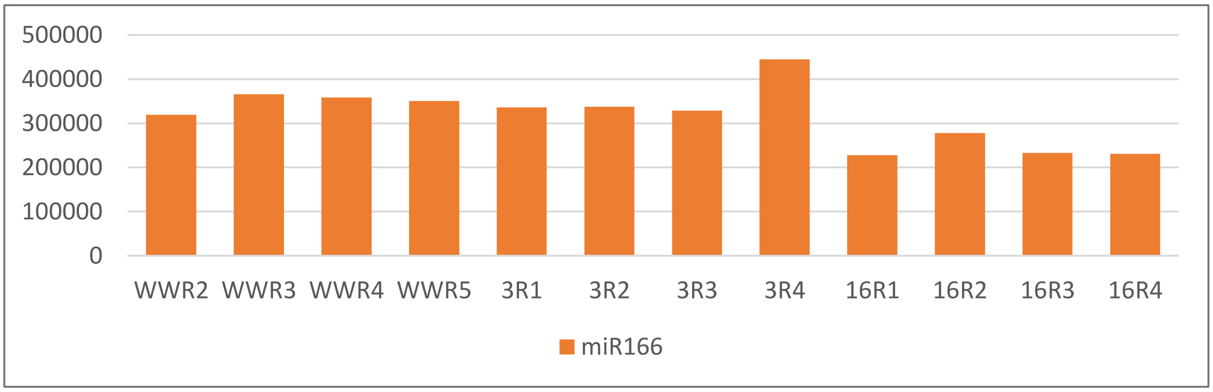

Supplement: Supplementary file 10 — Additional file 10: Figure S2.The RPTM normalized values for all miRNA belonging to the same miRNA family in each sample were summed. High abundance (> 100,000 average RPTM per sample), moderate abundance (> 1000 to < 20,000 average RPTM per sample), and low abundance (< 1000 average RPTM per sample) miRNA families are grouped together [file 12870_2019_2037_MOESM10_ESM.pdf]
