# Supplementary material for: Water-deficit responsive microRNAs in the primary root growth zone of maize
Source: BMC Plant Biol. 2019 Oct 24;19:447. doi: 10.1186/s12870-019-2037-y (PMC6814125; doi:10.1186/s12870-019-2037-y)
Supplement: Supplementary file 11 — Additional file 11: Figure S3. Plots of correlation between RNA-seq and stem-loop RT-qPCR results for the (A) -0.3 MPa vs WW, and (B) -1.6 MPa vs WW comparisons. The line of best fit is shown and the Spearman’s correlation coefficient (r) and p-values (p) are indicated on each plot [file 12870_2019_2037_MOESM11_ESM.pdf]

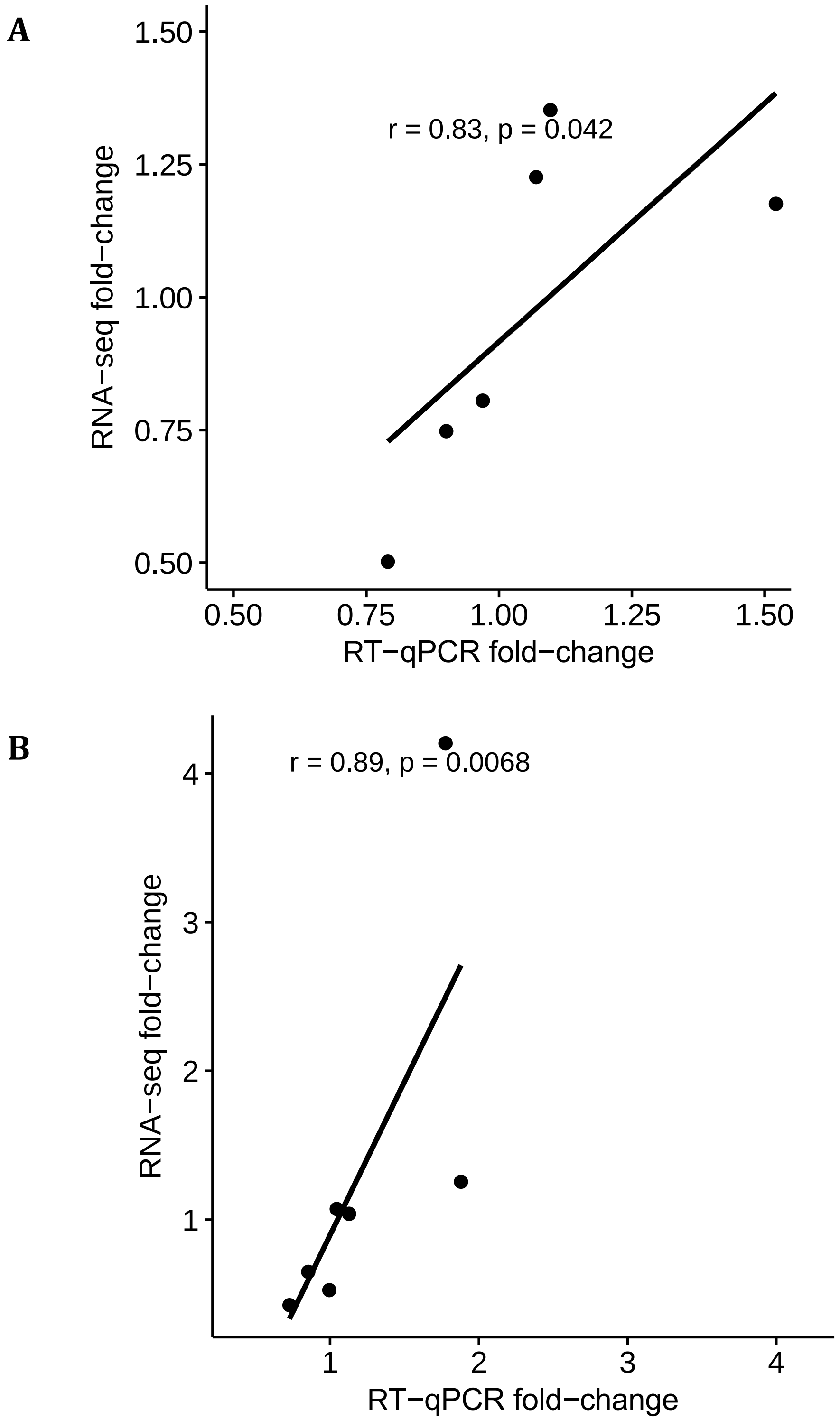

Figure S3. Plots of correlation between RNA-seq and stem-loop RT-qPCR results for the **(A)** -0.3 MPa vs WW, and **(B)** -1.6 MPa vs WW comparisons. The line of best fit is shown and the Spearman's correlation coefficient (r) and p-values (p) are indicated on each plot.
